# Preoperative HALP Profiles in Serous and Mucinous Epithelial Ovarian Tumors: Positioning Borderline Tumors on the Benign–Malignant Spectrum

**DOI:** 10.3390/jcm15197662

**Published:** 2026-10-03

**Authors:** Hanne Bulat Cim, Nisan Helin Dönmez, Melis Altuğ Inan, Fatma Ceren Gurel, Başak Cantürk Murt, Neslişah Ar, Ergül Demircivi, Abdulkadir Turgut

**Affiliations:** 1 Department of Obstetrics and Gynecology, Göztepe Prof. Dr. Süleyman Yalçın City Hospital, Istanbul 34722, Türkiye; nisanuzulmez@hotmail.com (N.H.D.); melaltug@gmail.com (M.A.I.); 2 Department of Obstetrics and Gynecology, Faculty of Medicine, Istanbul Medeniyet University, Istanbul 34700, Türkiye; drcerengurel@gmail.com (F.C.G.); basakcanturk2209@gmail.com (B.C.M.); neslisahar99@gmail.com (N.A.); drergul@yahoo.com (E.D.); abdulkadirturgut@gmail.com (A.T.)

**Keywords:** borderline ovarian tumor, epithelial ovarian cancer, HALP score, immunonutritional status, risk of malignancy index

## Abstract

**Background/Objectives:** Borderline ovarian tumors can overlap with benign and malignant adnexal masses in their preoperative features. The hemoglobin–albumin–lymphocyte–platelet (HALP) score reflects inflammatory and immunonutritional status. We compared HALP profiles across benign, borderline, and malignant epithelial ovarian tumors and secondarily explored their contribution beyond the risk of malignancy index (RMI). **Methods:** This retrospective single-center study included 464 women treated surgically for serous or mucinous epithelial ovarian tumors between 2015 and 2025: 164 benign cystadenomas, 78 borderline tumors, and 222 invasive carcinomas. HALP was compared among groups with additional adjustment for age and menopausal status. Regression and model-performance analyses were performed only in the borderline and malignant groups (*n* = 300). **Results:** Median HALP was lower in malignant tumors [28.6 (IQR 22.4)] than in benign [38.3 (23.8)] and borderline tumors [40.5 (21.4)] (both *p* < 0.001), whereas benign and borderline tumors were similar (*p* = 0.948). After adjustment, HALP remained lower in malignant tumors than in benign (adjusted median difference, −10.49; 95% CI, −15.47 to −4.55) and borderline tumors (−11.86; 95% CI, −18.63 to −5.84). In exploratory malignant-versus-borderline modeling, HALP was associated with malignant histology alone, but this association was attenuated after accounting for age and log-transformed RMI; adding HALP did not significantly improve model fit or discrimination. **Conclusions:** Preoperative HALP was consistently lower in malignant tumors, while borderline tumors resembled benign lesions. HALP may reflect systemic immunonutritional differences across pathological groups. Its incremental predictive role beyond established preoperative measures remains uncertain and requires external validation.

## 1. Introduction

Borderline ovarian tumors comprise epithelial neoplasms with biological features between benign cystadenomas and invasive ovarian carcinomas. They show epithelial proliferation and cytological atypia but lack destructive stromal invasion and represent approximately 10–20% of epithelial ovarian tumors [1,2,3]. Although their prognosis is substantially more favorable than that of invasive epithelial ovarian cancer, borderline tumors remain clinically important because they occupy a diagnostic and biological position between benign and malignant ovarian disease [4]. They also commonly affect women of reproductive age, making fertility preservation an important consideration in surgical planning [1]. Accordingly, their management requires accurate oncologic assessment while avoiding unnecessary radical procedures whenever clinically appropriate.

Distinguishing borderline tumors from benign lesions and invasive ovarian malignancies before surgery can be difficult. Ultrasonographic features and serum tumor markers frequently overlap across these pathological categories, while definitive diagnosis generally relies on postoperative histopathological examination [5,6]. Intraoperative frozen section analysis may also be inconclusive or discordant with final pathology, particularly in large or heterogeneous tumors, potentially complicating decisions regarding staging and fertility-sparing surgery [7]. These diagnostic overlaps provide a rationale for investigating systemic markers across the pathological spectrum of epithelial ovarian tumors.

Cancer-related systemic changes may involve inflammatory activity, anemia, altered immune function and nutritional impairment. The HALP score integrates hemoglobin (g/L), serum albumin (g/L), lymphocyte count (10^9^/L), and platelet count (10^9^/L), thereby reflecting both nutritional and systemic inflammatory status [8]. Its components collectively reflect anemia, nutritional status, immune status, and platelet-related inflammation. Low HALP scores have been associated with adverse clinicopathological characteristics and poorer outcomes in several solid malignancies, including cervical, endometrial, and ovarian cancers [9,10,11]. The preoperative HALP profile across benign, borderline, and malignant serous and mucinous epithelial ovarian tumors remains insufficiently characterized.

In this study, we compared preoperative HALP profiles across benign, borderline, and malignant serous and mucinous epithelial ovarian tumors. As a secondary objective, we evaluated whether HALP provided additional information beyond the RMI specifically for distinguishing malignant from borderline tumors.

## 2. Materials and Methods

### 2.1. Study Design and Population

This retrospective, single-center cohort study included patients who underwent surgery for an ovarian or adnexal mass at the Department of Obstetrics and Gynecology, Göztepe Prof. Dr. Süleyman Yalçın City Hospital. The study was conducted in accordance with the principles of the Declaration of Helsinki and was approved by the institutional ethics committee (approval no. 2025/0338; 18 December 2025).

Patients who underwent surgery between 1 January 2015, and 1 December 2025, following the identification of an ovarian or adnexal mass on preoperative imaging were screened for eligibility. According to the final histopathological diagnosis, eligible patients were classified into three groups: benign serous or mucinous ovarian cystadenomas, serous or mucinous borderline ovarian tumors, and primary invasive serous or mucinous ovarian carcinomas. All consecutive patients undergoing surgery during the predefined study period who met the eligibility criteria were screened for inclusion. The primary outcome was the difference in preoperative HALP levels among benign, borderline, and malignant ovarian tumors. Secondary exploratory analyses evaluated the incremental contribution of HALP beyond RMI for malignant–borderline discrimination and its associations with clinicopathological characteristics.

### 2.2. Eligibility Criteria

Patients were eligible if they had undergone preoperative imaging and primary surgical treatment, had one of the prespecified final histopathological diagnoses, and had sufficient preoperative clinical and laboratory data for the study analyses. Laboratory measurements obtained within 7 days before surgery and before any oncological treatment or blood transfusion were used to calculate the inflammatory and immunonutritional indices.

Patients with non-serous or non-mucinous epithelial ovarian tumors, non-epithelial ovarian tumors, or ovarian metastases originating from another primary malignancy or those who had received neoadjuvant chemotherapy before surgery were excluded. To minimize the potential influence of conditions affecting hematological, inflammatory, or nutritional parameters, patients with active infection or febrile illness, chronic inflammatory or autoimmune disease, hematological disorders, concurrent malignancy, clinically significant hepatic or renal dysfunction or pregnancy were excluded. Additional exclusion criteria were blood transfusion before laboratory sampling, systemic corticosteroid or immunosuppressive therapy, and anticoagulant or antiplatelet treatment because of their potential effects on platelet-related hematologic parameters and platelet-based inflammatory indices. Benign adnexal conditions associated with a substantial inflammatory response, including endometriomas and tubo-ovarian abscesses, were not included. Patients with missing essential clinical or laboratory data, including data required to calculate HALP or RMI, were excluded. No imputation was performed, and there were no missing HALP or RMI values in the final analytic cohort.

Histopathological diagnoses and pathological variables were obtained from the original final pathology reports and reflected the WHO classification in use at the time of diagnosis (4th edition, 2014, or 5th edition, 2020, as applicable). Archived specimens were not centrally rereviewed or uniformly reclassified according to a single WHO edition. Serous carcinomas were categorized as high-grade or low-grade according to the final pathology report. Primary mucinous ovarian carcinomas were included only when the final histopathological diagnosis supported an ovarian primary and there was no clinical, radiological, or pathological evidence of a non-ovarian primary malignancy; metastatic tumors to the ovary were excluded.

### 2.3. Data Collection and Variable Definitions

Demographic, clinical, laboratory, imaging, surgical, and histopathological data were retrospectively obtained from the hospital information management system and patient records using a standardized data collection form. Age, menopausal status, preoperative laboratory parameters, final histopathological diagnosis, and surgical characteristics were recorded.

For patients with borderline ovarian tumors, histological subtype, FIGO stage, presence of implants, microinvasion, micropapillary pattern, lymphovascular space invasion, completeness of surgical staging, recurrence, and the specific fertility-sparing procedure were additionally recorded from the original pathology and operative records. LVSI was defined as tumor cells or aggregates within morphologically identifiable lymphatic or vascular spaces. In the two LVSI-positive borderline cases, D2-40 staining performed during routine diagnostic workup supported the lymphatic nature of these spaces. Recurrence was defined as the detection of a new ovarian or extraovarian lesion consistent with borderline ovarian tumor or invasive carcinoma after primary surgery, documented by imaging and/or histopathological examination during follow-up. For patients with invasive ovarian carcinoma, histological subtype, serous tumor grade, lymphovascular space invasion, FIGO stage, and surgical approach were recorded.

### 2.4. Calculation of Hematological and Immunonutritional Indices

The hematological and immunonutritional indices were calculated using routine laboratory measurements obtained within 7 days before surgery.

Complete blood count parameters were measured using BC-6800 Plus hematology analyzer (Shenzhen Mindray Bio-Medical Electronics Co., Ltd., Shenzhen, China), serum albumin using the cobas c 8000 system (Roche Diagnostics GmbH, Mannheim, Germany), and serum CA-125 by electrochemiluminescence immunoassay (ECLIA) using the cobas e 602 analyzer (Roche Diagnostics GmbH, Mannheim, Germany).

The hemoglobin–albumin–lymphocyte–platelet score was calculated as follows:

HALP score = [hemoglobin (g/L) × albumin (g/L) × lymphocyte count (10^9^/L)]/platelet count (10^9^/L)

The prognostic nutritional index was calculated as follows:

PNI = 10 × serum albumin (g/dL) + 0.005 × total lymphocyte count (/mm^3^)

The neutrophil-to-lymphocyte ratio was calculated by dividing the absolute neutrophil count by the absolute lymphocyte count, and the platelet-to-lymphocyte ratio was calculated by dividing the platelet count by the absolute lymphocyte count.

The risk of malignancy index (RMI) was calculated using the classical formula: RMI = U × M × CA-125, where U denotes the ultrasound score, M the menopausal-status score and CA-125 the serum CA-125 concentration. The ultrasound score was based on five features: multilocularity, solid components, bilateral lesions, ascites, and suspected intra-abdominal metastasis. U was assigned as 0 when none of these features was present, 1 when one feature was present, and 3 when two or more features were present. The menopausal-status score was assigned as M = 1 for premenopausal women and M = 3 for postmenopausal women [12].

### 2.5. Surgical Procedures and Histopathological Evaluation

Surgical management was individualized according to the preoperative clinical and imaging findings, intraoperative assessment, suspected pathological category, and the patient’s fertility preferences. In patients with a suspected borderline or malignant ovarian tumor, intraoperative frozen-section examination was performed when considered clinically appropriate.

The extent of surgery was determined according to the frozen-section diagnosis, intraoperative findings, and the patient’s fertility preferences. Depending on the clinical and pathological characteristics of each case, surgical procedures included cystectomy, unilateral or bilateral salpingo-oophorectomy, or comprehensive staging surgery. Fertility-sparing surgery was defined as surgery preserving the uterus and at least one ovary or part of an ovary. The specific fertility-sparing procedure was determined from the operative records. Invasive ovarian carcinomas were staged according to the 2014 International Federation of Gynecology and Obstetrics (FIGO) staging system.

### 2.6. Statistical Analysis

All statistical analyses were performed using IBM SPSS Statistics, version 27.0, and R software, version 4.6.0. The distributions of continuous variables were assessed using the Shapiro–Wilk test and visual inspection. Continuous variables were presented as mean ± standard deviation or median with interquartile range, as appropriate, and categorical variables as numbers and percentages.

Comparisons among the benign, borderline, and malignant groups were performed using one-way analysis of variance or the Kruskal–Wallis test, as appropriate. When the overall comparison was statistically significant, pairwise comparisons were performed using Scheffé, Tamhane’s T2, or Dwass–Steel–Critchlow–Fligner post hoc tests according to data distribution and variance characteristics. Because HALP was right-skewed, adjusted comparisons among tumor groups were performed using median quantile regression with age and menopausal status as covariates. Pairwise comparisons were based on 1000 bootstrap resamples and adjusted using the Holm method. Exploratory subgroup comparisons of HALP were performed using the Mann–Whitney U test.

Recurrence-free survival was defined as the interval from primary surgery to documented recurrence; patients without recurrence were censored at the last available follow-up. Its association with HALP was assessed using an exploratory univariable Cox proportional hazards model, and the proportional hazards assumption was evaluated using Schoenfeld residuals.

For malignant-versus-borderline analyses, binary logistic regression was performed with borderline ovarian tumors as the reference category. Functional forms of continuous predictors were assessed using restricted cubic splines, and multicollinearity was evaluated using variance inflation factors and tolerance values. Because RMI was markedly right-skewed, it was natural-log transformed; age was modeled using a restricted cubic spline with four knots, whereas HALP was retained as a linear predictor. Five sequential models were evaluated: Model 1 included HALP; Model 2, log-transformed RMI; Model 3, log-transformed RMI and HALP; Model 4, age and log-transformed RMI; and Model 5, age, log-transformed RMI, and HALP. No zero RMI values were present in the malignant–borderline cohort; therefore, no additional handling was required before log transformation.

Regression results were reported as coefficients, odds ratios, 95% confidence intervals, and *p* values. Incremental contributions were assessed using likelihood-ratio tests comparing nested models. Model fit was evaluated using McFadden’s pseudo-R^2^ and the Akaike information criterion. Model performance was assessed using AUC, calibration intercept and slope, Brier score, 1000-bootstrap internal validation, and decision-curve analysis. Differences in AUC between nested models were compared using DeLong’s test. All statistical tests were two-sided, and *p* < 0.05 was considered statistically significant.

No a priori sample-size calculation was performed because all consecutive eligible patients during the predefined study period were included; the sample size was therefore determined by the available eligible cases.

## 3. Results

**Figure 1:**
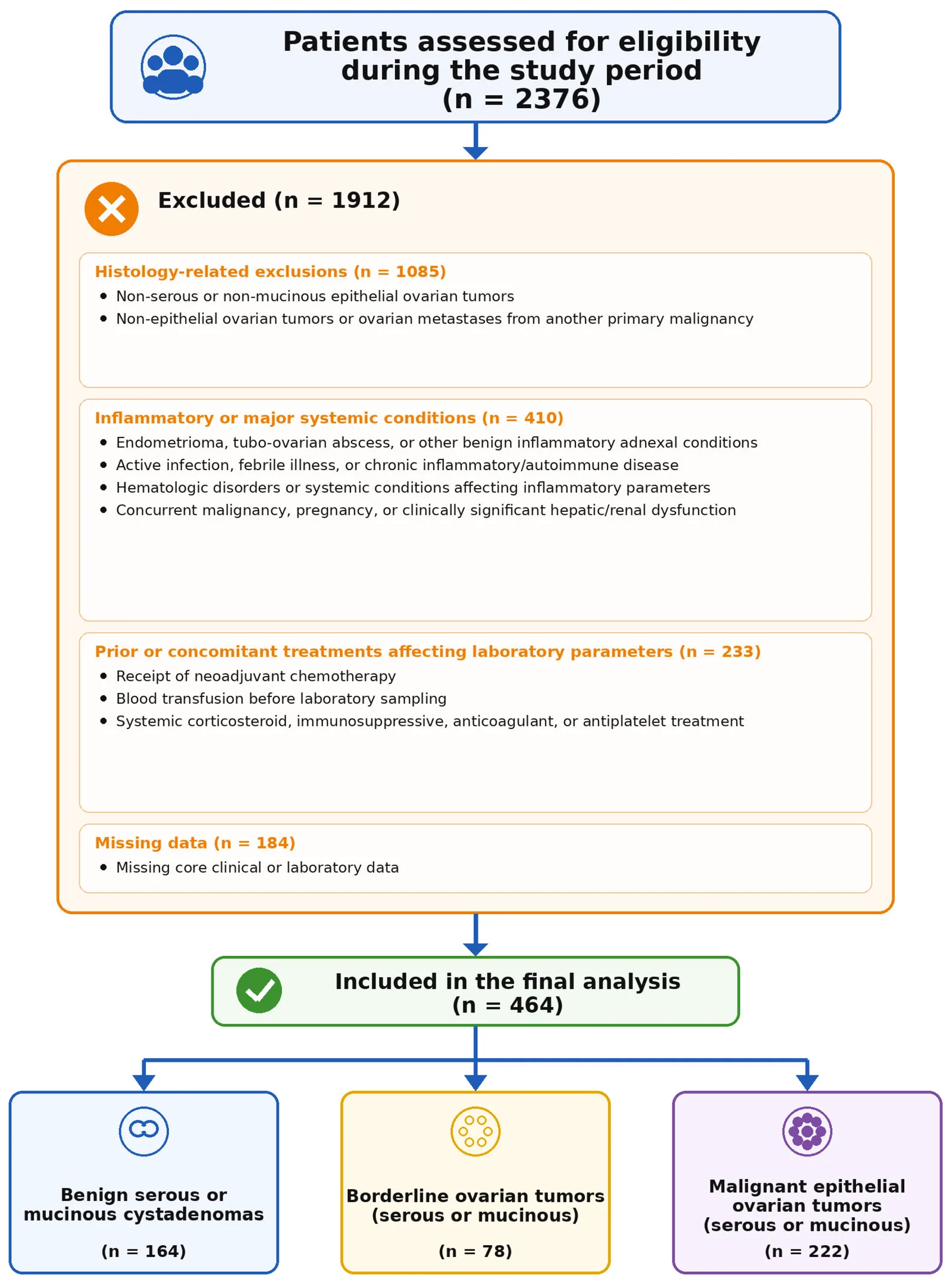
Flow diagram of patient selection.

A total of 2376 patients were screened, of whom 464 were included in the final analysis: 164 with benign serous or mucinous cystadenomas, 78 with borderline tumors, and 222 with invasive epithelial ovarian carcinomas (Figure 1).

**Figure 2:**
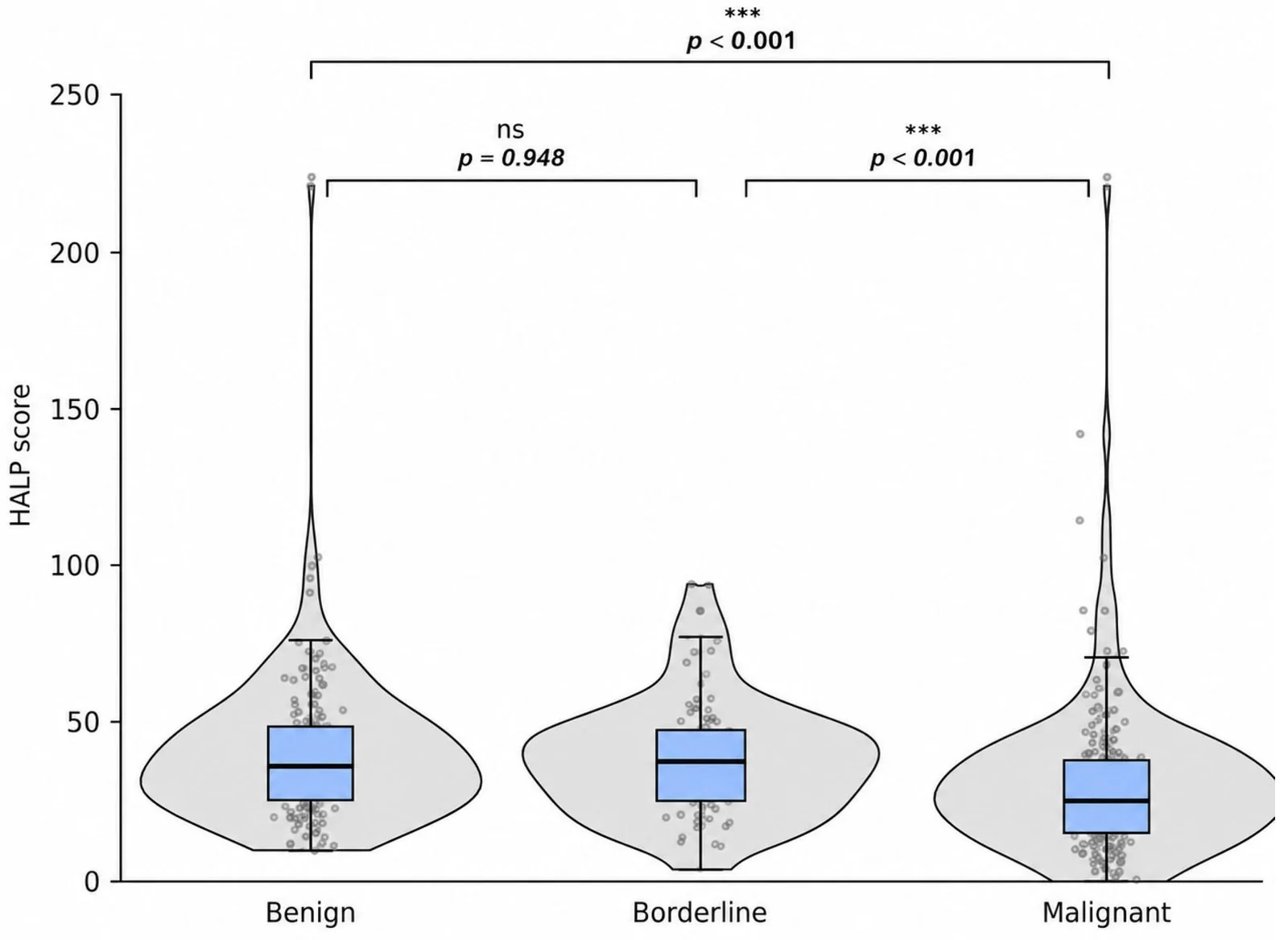
Distribution of preoperative HALP scores across benign, borderline, and malignant ovarian tumors. Violin plots show score distributions, with boxes indicating the median and interquartile range and dots representing individual patients. Pairwise comparisons are annotated in the figure. HALP, hemoglobin–albumin–lymphocyte–platelet score; *** *p* < 0.001; ns, not significant.

Baseline characteristics are summarized in Table 1. Patients in the malignant group were significantly older than those in the benign and borderline groups, whereas age did not differ significantly between the benign and borderline groups. HALP scores differed significantly across the three pathological groups (overall *p* < 0.001). Median HALP was lower in malignant tumors than in benign and borderline tumors (28.6 vs. 38.3 and 40.5, respectively; both *p* < 0.001), whereas no significant difference was observed between the benign and borderline groups (*p* = 0.948) (Table 2 and Figure 2). The malignant group also had lower PNI values and higher NLR, PLR, CA-125, and RMI values, while the borderline group had the largest median tumor diameter.

After adjustment for age and menopausal status, HALP remained lower in malignant tumors than in benign tumors (adjusted median difference, −10.49; 95 CI % bootstrap CI, −15.47 to −4.55) and borderline tumors (−11.86; 95% CI, −18.63 to −5.84; Holm-adjusted *p* < 0.001 for both). No significant difference was observed between benign and borderline tumors (+1.38; 95% CI, −3.98 to 7.41; *p* = 0.621).

Among the 78 patients with borderline ovarian tumors, 73 (93.6%) had FIGO stage I, three (3.8%) stage II, and two (2.6%) stage III disease. Implants were documented in five patients (6.4%), microinvasion in four (5.1%), and a micropapillary pattern in 11 (14.1%). Surgical staging was complete at initial surgery in 67 patients (85.9%) and incomplete in 11 (14.1%); four of the latter subsequently underwent restaging surgery. Fertility-sparing surgery was performed in 28 patients (35.9%): 10 underwent cystectomy-based surgery, 17 unilateral salpingo-oophorectomy, and one unilateral oophorectomy. LVSI was documented in two patients (2.6%); both were serous borderline tumors with microinvasion. Exploratory HALP comparisons showed no significant differences according to histological subtype or fertility-sparing surgery (Supplementary Table S1).

Median follow-up in the borderline group was 27 months (IQR 7–53.8), with eight recurrences occurring at a median of 24 months. In exploratory Cox analysis, HALP was not associated with recurrence-free survival (HR 1.006, 95% CI 0.966–1.047; *p* = 0.780), and the proportional hazards assumption was satisfied (Schoenfeld *p* = 0.32).

Among the 192 patients with serous ovarian carcinoma, 151 (78.6%) had high-grade and 41 (21.4%) low-grade serous carcinoma. In exploratory subgroup analyses, malignant tumors with LVSI had lower HALP scores than those without LVSI (*p* = 0.047); however, this finding was considered exploratory because of the multiple subgroup comparisons. HALP did not differ significantly according to histological subtype, FIGO stage, or surgical approach (Table 3).

In exploratory malignant-versus-borderline regression models, HALP was associated with malignant histology when evaluated alone, but this association was attenuated after accounting for log-transformed RMI and age (final-model *p* = 0.098). Adding HALP to the age- and RMI-based model did not significantly improve model fit (*p* = 0.114) (Table 4).

**Figure 3:**
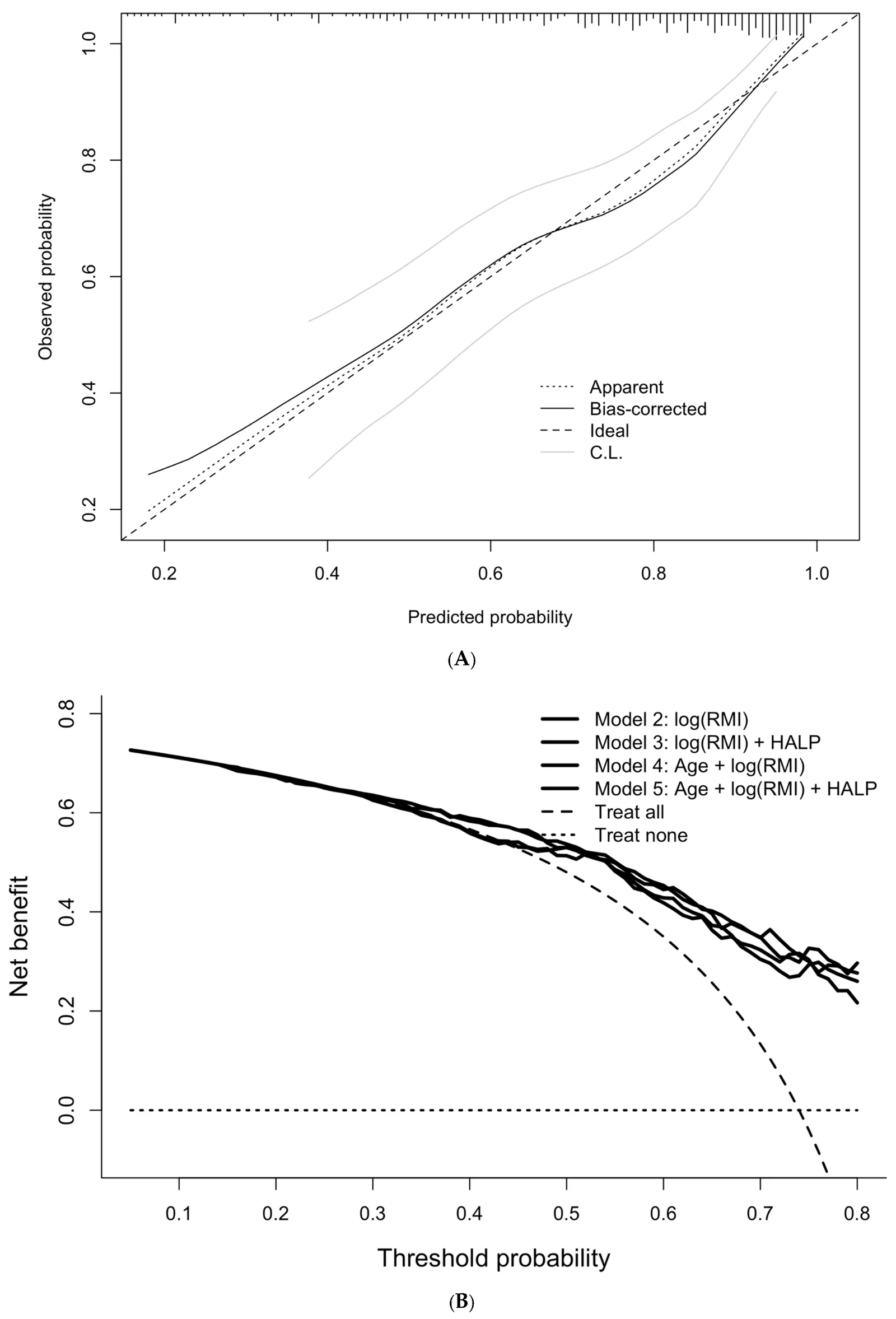
(**A**) Bootstrap bias-corrected calibration plot of the final multivariable model. Calibration plot for Model 5, incorporating age modeled with restricted cubic splines, log-transformed Risk of Malignancy Index (RMI), and hemoglobin–albumin–lymphocyte–platelet (HALP) score. Internal validation was performed using 1000 bootstrap resamples in 300 patients with borderline or invasive malignant ovarian tumors. The apparent and bootstrap bias-corrected calibration curves are shown together with the ideal 45° reference line and 95% confidence limits. (**B**) Decision curve analysis of the prediction models. Decision curves for Model 2 (log-transformed RMI), Model 3 (log-transformed RMI + HALP), Model 4 (age + log-transformed RMI), and Model 5 (age + log-transformed RMI + HALP) among 300 patients across threshold probabilities from 0.05 to 0.80.

Age showed evidence of nonlinearity (*p* = 0.0266), whereas HALP showed no significant departure from linearity (*p* = 0.1574); no residual nonlinearity was observed for ln(RMI) after transformation (*p* = 0.8102). No relevant multicollinearity was detected (VIF 1.031–1.119; tolerance 0.894–0.970). Adding HALP to the age- and RMI-based model changed the apparent AUC from 0.802 to 0.806, but this difference was not statistically significant (DeLong *p* = 0.453). The bootstrap-corrected AUC changed from 0.791 to 0.792. Calibration and Brier measures were similar between the two models, and decision-curve analysis showed broadly similar net-benefit profiles (Table 5 and Figure 3A,B).

## 4. Discussion

The principal finding of this study was that preoperative HALP values were consistently lower in malignant epithelial ovarian tumors than in benign and borderline tumors, whereas the latter two groups showed comparable values. This difference remained evident after adjustment for age and menopausal status. Although lower HALP values were also observed in LVSI-positive malignant tumors, this subgroup finding should be regarded as exploratory because of the multiple comparisons performed. In secondary malignant-versus-borderline analyses, HALP was associated with malignant histology when considered alone, but this association was attenuated after age and log-transformed RMI were included in the model.

HALP combines four routinely available parameters related to hematologic, nutritional, immune, and inflammatory status. Hemoglobin may be influenced by anemia and chronic inflammation, albumin by nutritional and inflammatory conditions, lymphocyte counts by immune status, and platelets by inflammatory and tumor-associated processes [10]. Consequently, a lower HALP score may reflect an unfavorable systemic profile, although none of its components is specific to malignancy [11]. In our cohort, malignant tumors were also associated with lower PNI and higher NLR and PLR values. The concordant direction of these indices supports the presence of broader systemic differences between the pathological groups. However, HALP may also be affected by age, anemia, nutritional status, inflammation, comorbidity, and overall disease burden, and should therefore not be interpreted as a tumor-specific biological marker.

The similarity in HALP profiles between the benign and borderline groups is also noteworthy. Borderline ovarian tumors occupy an intermediate histopathological position between benign cystadenomas and invasive carcinomas but lack destructive stromal invasion [13]. Although they may show epithelial proliferation, cytological atypia, microinvasion, or extraovarian implants, their clinical behavior differs from that of invasive carcinoma [14]. In our cohort, despite having the largest median tumor diameter, borderline tumors showed HALP values comparable to those of benign lesions, suggesting that tumor size alone does not explain the lower HALP observed in malignancy. These findings suggest that, despite their intermediate histopathological features, borderline tumors may resemble benign lesions more closely in terms of systemic HALP profile. Given the substantial overlap between benign and borderline tumors, HALP should not be used in isolation for preoperative discrimination; imaging findings, CA-125, RMI, and the overall clinical context remain essential for patient counseling and surgical planning.

Previous studies of hematological indices in ovarian masses have generally shown higher NLR and PLR values in malignant tumors than in benign or borderline lesions, while their ability to distinguish benign from borderline pathology appears limited [5]. Unlike these cell-based ratios, HALP also incorporates hemoglobin and albumin, thereby capturing hematologic and nutritional information in addition to inflammatory and immune-related parameters. Our findings extend this pattern to HALP, with lower values in malignant tumors and substantial overlap between the benign and borderline groups. The preoperative HALP profile across these three pathological categories remains insufficiently characterized. Previous ovarian HALP studies have mainly examined tumor stage and surgical outcomes [15,16]. By including borderline tumors within the same cohort, the present study provides comparative data across the benign–borderline–malignant spectrum.

An important secondary question was whether HALP contributed information beyond the RMI. The RMI combines menopausal status, ultrasonographic findings, and CA-125 and remains a practical tool for the preoperative assessment of ovarian masses [17]. HALP differs conceptually from the RMI by incorporating hematologic, inflammatory, immune, and nutritional parameters. In our malignant-versus-borderline analyses, HALP was associated with malignant histology when evaluated alone, but this association was attenuated after log-transformed RMI and age were incorporated into the models. Adding HALP to the age- and RMI-based model did not significantly improve model fit or discrimination. Thus, although HALP showed clear differences across the pathological groups, our data do not demonstrate a meaningful incremental predictive contribution beyond established preoperative measures. Further prospective and externally validated studies are needed to clarify whether HALP has any additional role in this setting.

In the borderline group, HALP was not associated with recurrence-free survival and did not differ according to histological subtype or fertility-sparing surgery. Because LVSI was present in only two borderline tumors, it was reported descriptively without inferential analysis. Previous studies suggest that recurrence in borderline ovarian tumors is more strongly related to established clinicopathological and surgical factors [18], while their histopathological and molecular heterogeneity is well recognized [13,19]. In the malignant group, LVSI-positive tumors had lower HALP values than LVSI-negative tumors (*p* = 0.047); however, this exploratory finding arose from multiple subgroup comparisons and should not be interpreted as evidence that HALP specifically reflects invasive tumor biology.

The strengths of this study include the evaluation of 464 patients with benign, borderline, and malignant serous and mucinous epithelial ovarian tumors within a single cohort. Restriction to these histological subtypes improved comparability across groups, and preoperative laboratory measurements were obtained within seven days before surgery. Patients with major conditions or treatments expected to substantially affect inflammatory or hematological parameters were excluded. HALP was also evaluated in relation to RMI rather than as an isolated biomarker. In addition, age- and menopausal status-adjusted group comparisons and expanded model-performance analyses allowed the principal and secondary study questions to be evaluated separately.

Several limitations should be considered. The retrospective, single-center design is susceptible to selection bias and residual confounding, and HALP may still be influenced by unrecognized anemia, nutritional or inflammatory conditions, comorbidity, and overall disease burden. Group sizes were unequal, and the predominance of serous carcinoma limited histology-specific inference, particularly for mucinous carcinoma. Only eight recurrences occurred in the borderline group, limiting the power of the time-to-event analysis, while the presence of LVSI in only two borderline tumors precluded meaningful inferential assessment. The malignant LVSI finding was also exploratory because of multiple subgroup comparisons. Histopathological data were derived from the original reports without central rereview or uniform reclassification according to a single WHO edition. Historical reagent-, assay-, or platform-level changes over the study period could not be fully reconstructed retrospectively and may have introduced some analytical variability. Although model performance was examined using discrimination, calibration, bootstrap internal validation, and decision-curve analysis, no external validation cohort was available. Finally, because the study included only surgically treated patients with final histopathological diagnoses, the findings may not be generalizable to all patients undergoing initial evaluation for an adnexal mass. Prospective multicenter studies with external validation are warranted.

## 5. Conclusions

Preoperative HALP scores were significantly lower in malignant epithelial ovarian tumors than in benign cystadenomas and borderline ovarian tumors, while no significant difference was observed between the benign and borderline groups. These differences persisted after adjustment for age and menopausal status. However, in secondary malignant-versus-borderline analyses, HALP did not provide a significant incremental predictive contribution beyond age and RMI. Thus, HALP may reflect systemic host differences across pathological groups, but its additional preoperative predictive role remains uncertain. Future prospective multicenter studies should use prespecified sample-size calculations based on the expected outcome frequency and model complexity and should include independent external validation of discrimination, calibration, and clinical net benefit.

## Figures and Tables

**Table 1:** Baseline demographic, clinical, ultrasonographic and tumor characteristics of patients ^†^.

	Benign Epithelial Ovarian Tumors (n = 164)	Borderline Epithelial Ovarian Tumors (n = 78)	Malignant Epithelial Ovarian Tumors (n = 222)
Age (years)	48.3 ± 14.5	49.7 ± 16.1	58.9 ± 12.4
Menopausal status			
No	55 (33.5)	28 (35.9)	30 (13.5)
Yes	109 (66.5)	50 (64.1)	192 (86.5)
Gravidity	2 (3)	2 (3.75)	3 (3)
Parity	2 (2)	1.5 (3)	2 (1)
Tumor diameter (cm)	7 (5)	9 (8)	7 (6.5)
Bilateral ovarian involvement (final diagnosis)			
No	146 (89.0)	66 (84.6)	164 (73.9)
Yes	18 (11.0)	12 (15.4)	58 (26.1)
Ultrasonographic multilocularity			
No	33 (20.1)	25 (32.1)	64 (28.8)
Yes	131 (79.9)	53 (67.9)	158 (71.2)
Ultrasonographic solid component			
No	123 (75.0)	49 (62.8)	49 (22.0)
Yes	41 (25.0)	29 (37.2)	173 (78.0)
Ultrasonographic bilateral involvement			
No	154 (93.9)	73 (93.6)	169 (76.1)
Yes	10 (6.1)	5 (6.4)	53 (23.9)
Ultrasonographic ascites			
No	164 (100)	78 (100)	174 (78.4)
Yes	0	0	48 (21.6)
Ultrasonographic suspicion of metastasis			
No	164 (100)	77 (98.7)	196 (88.3)
Yes	0	1 (1.3)	26 (11.7)

^†^ Data are presented as mean ± standard deviation, median (interquartile range), or number (%), as appropriate.

**Table 2:** Comparison of demographic, immunonutritional, inflammatory, and clinical parameters among benign, borderline, and malignant ovarian tumors.

**Panel A. Unadjusted Comparisons**					
**Variable**	**Group**	**Value**	**Overall *p*** **Value**	**Pairwise Comparison**	**Post Hoc** ***p*** **Value**
Age (years)	Benign	48.3 ± 14.5	<0.001	B vs. BL	0.931
	Borderline	49.7 ± 16.1		B vs. M	<0.001
	Malignant	58.9 ± 12.4		BL vs. M	<0.001
HALP	Benign	38.3 (23.8)	<0.001	B vs. BL	0.948
	Borderline	40.5 (21.4)		B vs. M	<0.001
	Malignant	28.6 (22.4)		BL vs. M	<0.001
PNI	Benign	52.2 (7.78)	<0.001	B vs. BL	0.053
	Borderline	54.5 (6.45)		B vs. M	<0.001
	Malignant	49.7 (8.48)		BL vs. M	<0.001
Albumin (g/L)	Benign	42.0 (7.05)	<0.001	B vs. BL	0.034
	Borderline	44.0 (3.95)		B vs. M	0.353
	Malignant	42.0 (6.05)		BL vs. M	<0.001
Risk of Malignancy Index (RMI)	Benign	37.6 (43.4)	<0.001	B vs. BL	<0.001
	Borderline	78.5 (211.0)		B vs. M	<0.001
	Malignant	560.0 (2380.0)		BL vs. M	<0.001
Neutrophil-to-lymphocyte ratio (NLR)	Benign	2.00 (0.93)	<0.001	B vs. BL	0.215
	Borderline	2.20 (1.27)		B vs. M	<0.001
	Malignant	2.73 (2.24)		BL vs. M	0.027
Platelet-to-lymphocyte ratio (PLR)	Benign	139 (68.8)	<0.001	B vs. BL	0.978
	Borderline	136 (64.9)		B vs. M	<0.001
	Malignant	161 (128.0)		BL vs. M	0.001
Tumor diameter (cm)	Benign	7 (5)	<0.001	B vs. BL	<0.001
	Borderline	9 (8)		B vs. M	0.856
	Malignant	7 (6.5)		BL vs. M	<0.001
Preoperative CA-125 (IU/mL)	Benign	15.2 (15.9)	<0.001	B vs. BL	<0.001
	Borderline	32.2 (64.0)		B vs. M	<0.001
	Malignant	91.2 (412.0)		BL vs. M	<0.001
**Panel B. Age and Menopausal Status-Adjusted HALP Comparisons**					
**Comparison**		**Adjusted Median Difference**		**95% Bootstrap CI**	**Holm-Adjusted *p* Value**
Borderline vs. benign		+1.38		−3.98 to 7.41	0.621
Malignant vs. benign		−10.49		−15.47 to −4.55	<0.001
Malignant vs. borderline		−11.86		−18.63 to −5.84	<0.001

Data are presented as mean ± standard deviation or median (interquartile range), as appropriate. Overall comparisons were performed using one-way analysis of variance or the Kruskal–Wallis test, according to data distribution. Pairwise comparisons were performed using Scheffé, Tamhane’s T2, or Dwass–Steel–Critchlow–Fligner post hoc tests, as appropriate. B, benign; BL, borderline; M, malignant; HALP, hemoglobin–albumin–lymphocyte–platelet score; PNI, prognostic nutritional index; RMI, risk of malignancy index; NLR, neutrophil-to-lymphocyte ratio; PLR, platelet-to-lymphocyte ratio; CA-125, cancer antigen 125. Adjusted median differences were estimated using median quantile regression with age and menopausal status as covariates. Confidence intervals were obtained from 1000 bootstrap resamples, and pairwise *p* values were adjusted using the Holm method.

**Table 3:** Comparison of preoperative HALP scores according to clinicopathological characteristics in patients with malignant ovarian tumors.

Variable	HALP, Median (IQR)	*p* Value
Histological subtype (n = 222)		
Serous (n = 192)	28.6 (22.8)	
Mucinous (n = 30)	30.3 (18.6)	0.700
Lymphovascular space invasion (n = 222)		
No (n = 187)	29.7 (21.8)	
Yes (n = 35)	24.6 (20.1)	0.047
FIGO stage (n = 222)		
Stage I–II (n = 66)	9.1 (20.3)	
Stage III–IV (n = 156)	28.4 (23.4)	0.678
Type of surgery		
Laparotomy (n = 216)	28.6 (22.3)	
Laparoscopy (n = 6)	30.7 (34.6)	0.709

Data are presented as median (interquartile range). Comparisons were performed using the Mann–Whitney U test. HALP, hemoglobin–albumin–lymphocyte–platelet score; IQR, interquartile range; LVSI, lymphovascular space invasion; FIGO, International Federation of Gynecology and Obstetrics.

**Table 4:** Logistic regression models evaluating HALP and RMI in the distinction between malignant and borderline ovarian tumors.

	Model 1: HALP	Model 2: log(RMI)	Model 3: log(RMI) + HALP	Model 4: Age + log(RMI)	Model 5: Age + log(RMI) + HALP
HALP, OR (95% CI)	0.983 (0.971–0.995)	—	0.991 (0.979–1.003)	—	0.990 (0.977–1.002)
*p* value	0.005	—	0.145	—	0.098
log(RMI), OR (95% CI)	—	1.782 (1.483–2.141)	1.743 (1.448–2.100)	1.650 (1.369–1.989)	1.609 (1.332–1.944)
*p* value	—	<0.001	<0.001	<0.001	<0.001
Age, overall *p* value	—	—	—	<0.001	<0.001
Model LR χ^2^ (df)	9.05 (1)	52.03 (1)	54.02 (2)	71.42 (4)	73.92 (5)
Model *p* value	0.003	<0.001	<0.001	<0.001	<0.001
McFadden R^2^	0.026	0.151	0.157	0.208	0.215
AIC	338.79	295.80	295.82	282.41	281.91

Logistic regression models were fitted for malignant versus borderline ovarian tumors (n = 300), with borderline tumors as the reference category. RMI was natural-log transformed, and age was modeled using a restricted cubic spline with four knots. The overall *p* value for age represents the joint test of the spline terms. Model LR χ^2^ represents the likelihood-ratio test comparing each fitted model with the intercept-only model, whereas incremental contributions were assessed using likelihood-ratio tests between nested models. The full coefficient specification of Model 5, including the intercept and spline coefficients, is provided in Supplementary Table S2. AIC, Akaike information criterion; HALP, hemoglobin–albumin–lymphocyte–platelet score; LR, likelihood ratio; OR, odds ratio; RMI, Risk of Malignancy Index.

**Table 5:** Predictive performance and internal validation of logistic regression models for malignant versus borderline ovarian tumors.

**A. Model Performance and Internal Validation**					
**Model**	**95% CI**	**Bootstrap-Corrected AUC**	**Calibration Intercept**	**Calibration Slope**	**Brier Score**
Model 1: HALP	0.681 (0.615–0.746)	0.681	−0.232	1.207	0.191
Model 2: log(RMI)	0.766 (0.707–0.826)	0.765	−0.003	1.009	0.162
Model 3: log(RMI) + HALP	0.770 (0.712–0.829)	0.768	0.000	0.987	0.163
Model 4: Age + log(RMI)	0.802 (0.748–0.856)	0.791	0.048	0.933	0.155
Model 5: Age + log(RMI) + HALP	0.806 (0.754–0.859)	0.792	0.068	0.913	0.156
**B. Incremental Discrimination**					
**Comparison**	**ΔAUC**	**Correct 95% CI**		***p***	
Model 2 → Model 3	+0.004	−0.007 to 0.015		0.491	
Model 2 → Model 4	+0.036	−0.001 to 0.073		0.060	
Model 3 → Model 5	+0.036	−0.001 to 0.073		0.054	
Model 4 → Model 5	+0.004	−0.007 to 0.016		0.453	

AUC, area under the receiver operating characteristic curve; Brier, Brier score; CI, confidence interval; HALP, hemoglobin–albumin–lymphocyte–platelet score; RMI, Risk of Malignancy Index. Models were fitted in 300 patients with borderline or invasive malignant ovarian tumors (78 borderline and 222 malignant tumors). Age was modeled using a restricted cubic spline with four knots, and RMI was log-transformed because of marked right-skewness. Internal validation was performed using 1000 bootstrap resamples. Bootstrap-corrected AUC was derived from the optimism-corrected Somers’ Dxy statistic. Calibration intercept, calibration slope, and Brier score are optimism-corrected estimates. Differences in apparent AUC between nested models were assessed using paired DeLong tests.

## Data Availability

The data presented in this study are available from the corresponding author upon reasonable request. The data are not publicly available because of patient privacy and institutional ethical restrictions.

## Supplementary Materials

The following supporting information can be downloaded at: https://www.mdpi.com/article/10.3390/jcm15197662/s1, Table S1: Comparison of preoperative HALP scores according to clinicopathological characteristics in patients with borderline ovarian tumors; Table S2: Full coefficient specification of the final Model 5.
