# Supplementary material for: Preoperative HALP Profiles in Serous and Mucinous Epithelial Ovarian Tumors: Positioning Borderline Tumors on the Benign–Malignant Spectrum
Source: J Clin Med. 2026 Oct 3;15(19):7662. doi: 10.3390/jcm15197662 (PMC13665774; doi:10.3390/jcm15197662)
Supplement: Supplementary file 1 [file jcm-15-07662-s001.zip › jcm-4473070-supplementary.pdf]

Supplementary Table S1: **Comparison of preoperative HALP scores according to clinicopathological characteristics in patients with borderline ovarian tumors**

Variable	HALP, Median (IQR)	*P* value
**Histological subtype (n=78)**		
**Serous (n=45)**	37.0 (22.6)	
**Mucinous (n=33)**	43.2 (18.8)	0.189
**Fertility-sparing surgery (n=78)**		
**No (n=50)**	39.6 (23.2)	
**Yes (n=28)**	42.8 (16.8)	0.947

Data are presented as median (interquartile range). Comparisons were performed using the Mann–Whitney U test. HALP: hemoglobin–albumin–lymphocyte–platelet score.

Supplementary Table S2. Full coefficient specification of the final Model 5

Parameter	β coefficient
Intercept	−3.2915
Age	0.0325
Age′	0.1051
Age″	−0.7681
ln(RMI)	0.4755
HALP	−0.0105

The final model equation was:
logit[P(malignant)] = −3.2915 + 0.0325(Age) + 0.1051(Age′) − 0.7681(Age″) + 0.4755×ln(RMI) − 0.0105×HALP. Age was modeled using restricted cubic splines with four knots at 32.95, 52.00, 62.00, and 79.05 years. Age′ and Age″ represent the nonlinear spline basis functions. RMI was natural-log transformed.
